# Nitrogen supply rate regulates microbial resource allocation for synthesis of nitrogen-acquiring enzymes

**DOI:** 10.1371/journal.pone.0202086

**Published:** 2018-08-14

**Authors:** Kazuki Fujita, Takashi Kunito, Junko Matsushita, Kaori Nakamura, Hitoshi Moro, Seishi Yoshida, Hideshige Toda, Shigeto Otsuka, Kazunari Nagaoka

**Affiliations:** 1 Department of Environmental Sciences, Faculty of Science, Shinshu University, Matsumoto, Japan; 2 Nagano Prefecture Vegetable and Ornamental Crops Experiment Station, Shiojiri, Japan; 3 Department of Applied Biological Chemistry, Graduate School of Agricultural and Life Sciences, The University of Tokyo, Bunkyo-ku, Tokyo, Japan; 4 Central Region Agricultural Research Center, NARO, Tsukuba, Japan; RMIT University, AUSTRALIA

## Abstract

Although microorganisms will preferentially allocate resources to synthesis of nitrogen (N)-acquiring enzymes when soil N availability is low according to the resource allocation model for extracellular enzyme synthesis, a robust link between microbial N-acquiring enzyme activity and soil N concentration has not been reported. To verify this link, we measured several indices of soil N availability and enzyme activity of four N-acquiring enzymes [*N*-acetyl-β-glucosaminidase (NAG), protease (PR), urease (UR), and L-asparaginase (LA)] and a carbon (C)-acquiring enzyme [β-D-glucosidase (BG)] in arable and forest soils. Although the ratios of NAG/BG and PR/BG were not significantly related with indices of soil N availability, ratios of LA/BG and UR/BG were strongly and negatively related with potentially mineralizable N estimated by aerobic incubation but not with pools of labile inorganic N and organic N. These results suggest that microorganisms might allocate their resources to LA and UR synthesis in response to N supply rate rather than the size of the easily available N pools. It was also suggested that the underlying mechanism for synthesis was different between these N-acquiring enzymes in soil microorganisms: microbial LA and UR were primarily synthesized to acquire N, whereas NAG and PR syntheses were regulated not only by N availability but also by other factors.

## Introduction

Although gains in ecosystem N are made through molecular N_2_ fixation by microorganisms and N deposition from the atmosphere, the amounts are usually low, resulting in widespread N limitation to primary production in many terrestrial and aquatic ecosystems [1]. Therefore, microbial decomposition of plant litter and soil organic matter plays an important role in N supply to plants and microorganisms in terrestrial ecosystems. Microorganisms acquire N through decomposing organic N to ammonium (NH_4_^+^) or assimilable small organic N (e.g., amino acids) by various enzymes [2]. However, the underlying mechanism of microbial regulation of N-acquiring enzyme synthesis in response to N availability in soils is not well understood.

According to the resource allocation model for extracellular enzyme synthesis [3, 4], microorganisms will preferentially allocate their resources to enzymes for acquiring an element limiting their productivity. Moro et al. [5] and Fujita et al. [6] found that the resource allocation for phosphatase synthesis depends on labile inorganic phosphorus (P) level across various soils. In contrast to the P-acquiring enzymes with a demonstrated robust link to microbial P demand, the correspondence between N-acquiring enzyme activity and N demand is weak because mineralization of organic N compounds is often coupled to energy acquisition [3, 7–9]. A meta-analysis showed that N fertilization did not affect N-acquiring enzyme activities in soils [10]. Sinsabaugh et al. [11] suggested that a very weak negative correlation between β-D-glucosidase/(*N*-acetyl-β-glucosaminidase + leucine aminopeptidase) ratio [BG/(NAG + LAP) ratio] and total C/total N ratio (*R*^2^ = 0.03; *n* = ca. 2200) found in freshwater sediments was ascribable to the microbial synthesis of NAG and LAP to acquire not only N but also C. Recently, however, Moro et al. [5] reported a strong negative relationship between the ratio of N-acquiring enzyme [L-asparaginase (LA)] to C-acquiring enzyme (BG) activities and total N concentration in an agricultural field that had been subjected to different fertilization managements. A significant correlation was also found between BG/(NAG + LAP) ratio and mineralizable N in wet tropical forest soils [12]. These inconsistencies among the published reports might have occurred because of the ignorance of existing forms of soil N (i.e., availability) and differences between N-acquiring enzymes in the response of their synthesis to N availability in soils. The objectives of the present study were (1) to examine which soil N form and/or N availability index microorganisms respond to when investing their resources in N-acquiring enzyme synthesis and (2) to determine whether synthesis of four N-acquiring enzymes respond similarly to N availability in arable and forest soils.

## Materials and methods

### Soils

Arable Andosol samples were collected from the Ap horizon in an experimental field at the Nagano Prefecture Vegetable and Ornamental Crops Experiment Station in Shiojiri, Nagano Prefecture, Japan [6]. The field experiment had been under 14 different fertilization management systems since 1967 (S1 Table). After their collection in June 2004, the soil samples were immediately sieved through a 2-mm mesh and air-dried. Then, the samples were stored at room temperature until their use in this experiment in 2014. Celery was harvested for each fertilization management system in November 2004. Nitrogen content in the celery was determined after digestion with sulfuric acid and hydrogen peroxide in a heated block.

We also collected arable Andosols (*n* = 21) in Ibaraki, Nagano, Niigata, Kumamoto, Tokyo, Hokkaido, and Miyagi Prefectures, and arable Acrisols (*n* = 3) and Fluvisols (*n* = 3) from Ibaraki Prefecture, Japan. Sixteen soil samples collected in 2004 were air-dried and used in this study. The other eleven soil samples collected in 2016 were stored at −20°C immediately after sampling for enzyme analysis, and aliquots of the samples were air-dried for chemical analyses.

Forest soil samples were collected from the A horizon in forests in Nagano Prefecture, Japan in 2004 [6]. Twenty-two soils were classified as Cambisols, and seven soils as Andosols. After sieving through a 2-mm mesh and air-drying, the samples were stored at room temperature until their use in this experiment in 2014.

Soil pH was measured from a soil–water suspension of 1:2.5 (w/v). Soil total C and total N were measured with an NC analyzer (Yanaco MT-5, Kyoto, Japan).

### Chemical indices of nitrogen availability

Exchangeable NH_4_^+^ and nitrate (NO_3_^−^) were extracted from soils using 2 M KCl, and the concentrations of these ions (KCl-IN) were determined by the steam distillation method with MgO and Devarda’s alloy [13]. Total N in the KCl extract (KCl-TN) was also determined by oxidation with peroxodisulfate in an autoclave at 120°C for 30 min, followed by ultraviolet (UV) spectrophotometric determination of NO_3_^−^ [14]. Organic N in the KCl extract (KCl-ON) was calculated as the difference between KCl-TN and KCl-IN. KCl-IN is readily available for utilization by soil microorganisms [15].

Autoclave extraction was conducted by autoclaving soil for 1 h at 110°C in 2 M KCl solution [16, 17], and total N in the autoclave extracts (autoclave-TN) was determined using the UV spectrophotometric method after alkaline peroxodisulfate digestion as described above. Autoclaving kills microorganisms, and therefore the extracts contain N released from dead microorganisms. Also, some of the soil organic matter can be hydrolyzed during autoclaving [16]. Saito [17] reported that autoclave-TN had a strong correlation with the amount of potentially mineralizable N estimated by the aerobic incubation method.

The phosphate buffer extraction method was originally developed to extract proteins from soils [18]. Phosphate buffer-extractable organic N (PEON) is frequently used as an index of available organic N in arable soils of Japan. We extracted N from soils using 0.067 M phosphate buffer (pH 7.0) as described elsewhere [19, 20]. Inorganic N (PEIN) and total N (PETN) in the phosphate buffer extracts were determined as described above, and PEON was calculated as the difference between PETN and PEIN.

NaHCO_3_-extractable N was extracted using 0.01 M NaHCO_3_, and UV absorbances at 205 nm (UV-205) and 260 nm (UV-260) in the extract were measured to evaluate N availability according to Hong et al. [21] and Sharifi et al. [22] with a modification (1:50 (w/v) soil:solution ratio instead of 1:20). UV-205 represents both organic and inorganic N forms in the extract, while UV-260 corresponds to organic matter content in the extract [23]. It was reported that these indices had a strong relationship with the amount of potentially mineralizable N estimated by the aerobic incubation method [22].

### Incubation methods for nitrogen availability

The aerobic incubation method is the most prevalent method used for assessing N availability in soils, although there are many variations in incubation methodology [24]. In the present study, air-dried soil samples were moistened to 60% of water holding capacity, and were incubated at 30°C for 28 days in the dark [25]. After incubation, inorganic N was extracted with 2 M KCl, and the concentration was determined as described above. The inorganic N content in soils after incubation was designated as Aer-IN, and net N mineralization (i.e., the increase in inorganic N during the 28 day-incubation) was estimated by subtracting the initial inorganic N level from Aer-IN and was designated as Aer-N_min_.

The anaerobic incubation method is also known to provide a good representation of N availability in both arable and forest soils [26–28]. We conducted anaerobic incubation of soils under waterlogged condition at 40°C for 7 days according to Kandeler [29]. After incubation, exchangeable NH_4_^+^ was extracted with 2 M KCl and determined by a modified Berthelot reaction [30]. The increase in NH_4_^+^ after the 7 day-incubation was designated as Ana-N_min_.

### Soil enzyme activities

β-D-glucosidase (BG) was chosen as a representative C-acquiring enzyme, and *N*-acetyl-β-glucosaminidase (NAG), protease (PR), urease (UR) and L-asparaginase (LA) as N-acquiring enzymes. BG is involved in hydrolysis of cellobiose, the main product in hydrolysis of cellulose by cellulases [31]. NAG cleaves *N*-acetylglucosamine from the nonreducing end of chitin, a structural element in various organisms including fungi and insects [32]. PR involves the degradation of proteins, and UR catalyzes the hydrolysis of urea to NH_3_ and CO_2_. It should be noted that urea is extensively used in agriculture but is also produced as a degradation product of nucleic acids, and is thus an important organic N compound in the environment [33]. LA catalyzes the hydrolysis of L-asparagine and a variety of *N*-substituted asparagine analogues [34, 35].

BG activity was measured with *p*-nitrophenyl-β-D-glucopyranoside as the substrate in a modified universal buffer (MUB) at pH 6.0 [31]. NAG activity was measured with *p*-nitrophenyl-2-acetamido-2-deoxy-β-D-glucopyranoside as the substrate in a MUB at pH 5.5 according to Miah et al. [36] with some modifications (incubation temperature was modified to 37°C instead of 30°C, and without toluene). PR activity was measured with casein sodium as the substrate in a 0.05 M Tris buffer at pH 8.1 [37]. UR was measured with urea as the substrate in a borate buffer at pH 10 [38]. LA was measured with L-asparagine as the substrate in a phosphate buffer at pH 7.6 [39].

For air-dried soil samples, enzyme activities were measured after rewetting to 60% of the water holding capacity and then incubating for 1 week at 22°C. Air-dried soil samples were employed in this study because we aimed to evaluate whether the resource allocation model is applicable for evaluating N availability in soils, in the same way as is usually done for routine soil N tests. It should be emphasized that this study is not intended to estimate enzyme activities in the original moist soil before long-term storage, but the air-dried soil is treated as an independent sample in itself. The Birch effect did not seem to cause a significant problem in this study, because our preliminary results showed that the ratio of LA/BG activities increased slightly 3 days after rewetting in some soils but then it remained stable during incubation for 14 days (S1 Fig). It was also reported that enzyme activities in the rewetted soils were highly correlated with those in the original moist soils, although the values in the rewetted soils were lower than those in the original moist soils [40]. To assess the effect of storage conditions on the resource allocation model for enzyme synthesis, we also used moist soil samples stored at −20°C.

### Statistical analyses

The Welch–Aspin test was used to detect a significant difference in soil properties between soils. Spearman’s rank correlation coefficient, and standardized major axis and ordinary least squares regression analyses were used to measure the strength of the association between soil properties. A stepwise multiple linear regression analysis was conducted to investigate the soil properties influencing the LA/BG and UR/BG ratios. A *P* value of less than 0.05 was considered as indicating statistical significance. These analyses were done using R version 3.4.3 [41] and the R package “smatr” [42].

## Results

### Soil chemical properties and enzyme activities in arable Andosols from Shiojiri

We investigated the relationships between soil properties and enzyme activities in arable Andosols with different fertilization management treatments (*n* = 14) in an experimental field in Shiojiri. Chemical properties in the Shiojiri soils are shown in Table 1. Total C, total N, and available N levels increased with application rate of compost and chemical fertilizer in the soils. Spearman's rank correlation coefficients between various indices of N availability are shown in Table 2. Except for KCl-ON and phosphate buffer extractable N, all of the indices of N availability correlated significantly with each other. N uptake of celery (Yoshida, unpublished results) also correlated significantly with available N concentrations except for KCl-IN, KCl-ON, and phosphate buffer extractable N.

**Table 1:** Chemical properties of the soil used.

	Soil pH	Total C (mg g^−1^)	Total N (mg g^−1^)	KCl extraction		Aer-IN	Aer-N_min_	Ana-N_min_	Autoclave-TN	Phosphate buffer extraction			NaHCO_3_ exctraction	
				IN	ON					PEIN	PEON		UV-205	UV-260
				(μg N g^−1^)										
Arable Andosols in Nagano Prefecture Vegetable and Ornamental Crops Experiment Station at Shiojiri														
No fertilizer	6.9	42.0	2.81	28.1	98.8	38.2	10.1	4.5	82.9	11.0	77.1	0.27		0.12
PK	6.9	45.7	3.48	29.7	92.6	41.4	11.7	2.2	63.2	14.5	106	0.31		0.15
NK	6.2	37.4	2.36	31.4	114	37.0	5.6	5.2	63.7	8.8	77.7	0.19		0.09
NP	6.1	36.7	2.87	30.1	78.4	45.3	15.2	1.4	88.1	11.4	48.7	0.28		0.15
NPK	6.2	42.9	3.45	26.6	125	54.0	27.4	11.0	87.7	10.9	53.0	0.33		0.15
Low NPK	6.4	39.1	3.07	30.5	115	65.9	35.4	6.5	104	12.0	91.6	0.54		0.23
High NPK	6.2	41.5	2.87	29.8	86.2	57.0	27.2	7.7	105	9.5	52.7	0.45		0.25
Very high NPK	5.9	46.2	3.54	29.5	108	59.3	29.8	7.4	114	14.3	44.6	0.33		0.16
20 t compost ha^-1^	6.9	36.4	2.95	33.9	117	63.2	29.3	7.7	114	11.2	43.0	0.46		0.18
PK + 20 t compost ha^-1^	6.8	46.1	4.03	32.5	125	74.1	41.5	13.3	116	9.6	53.9	0.62		0.29
NK + 20 t compost ha^-1^	6.2	38.9	3.05	38.9	118	60.8	22.0	12.3	102	11.3	80.8	0.64		0.29
NP + 20 t compost ha^-1^	6.3	53.2	4.84	38.8	167	82.2	43.4	10.4	137	11.2	56.5	0.59		0.26
NPK + 20 t compost ha^-1^	6.4	54.7	4.35	35.7	195	93.3	57.5	19.6	215	21.1	84.8	0.68		0.21
NPK + 40 t compost ha^-1^	6.1	60.3	4.24	106	54.3	187	81.4	29.0	188	40.5	49.0	0.83		0.26
Arable soils from other sites														
Andosols*	6.1±0.7	53.2±22.8	4.3±1.6	127±123	NA	175±147	50.5±57.5	60.1±54.6	295±159	79±106	96.9±42.2	1.57±0.67		0.31±0.13
Acrisols (*n* = 3)	5.8±0.1	16.1±1.5	1.4±0.2	87.7±27.6	NA	110±28.2	22.7±4.4	20.1±3.9	134±34	73.9±16.5	45.0±16.4	2.09±0.29		1.04±0.25
Fluvisols (*n* = 3)	6.1±0.1	14.0±1.5	1.2±0.1	91.8±49.0	NA	103±25	21.9±28.6	26.9±9.1	122±35	64.5±49.1	53.0±7.1	1.20±0.50		0.33±0.04
Forest soils														
Cambisols (*n* = 22)	5.1±0.7	63.8±46.8	4.4±2.6	62.7±35.4	NA	204±118	142±93.7	54.6±31.6	260±179	36.1±21.0	123±33.2	2.11±0.54		0.39±0.17
Andosols (*n* = 7)	5.5±0.4	95.0±47.3	6.9±2.7	123±127	NA	336±115	213±83.2	87.1±28.3	148±97.8	56.9±60.0	144±31	1.70±0.40		0.31±0.10

*, *n* = 21 for soil pH, total C, total N, KCl-IN, KCl-ON, Aer-IN, and Aer-N_min_; *n* = 10 for other properties.

Aer-IN, extractable inorganic N after aerobic incubation; Aer-N_min_, potentially mineralizable N estimated by aerobic incubation; Ana-N_min_, potentially mineralizable N estimated by anaerobic incubation; PEIN, phosphate buffer-extractable inorganic N; PEON, phosphate buffer-extractable organic N; NA, not available.

**Table 2:** Spearman's rank correlation coefficients (*r*) among various indices of N availability in the arable Andosols at Shiojiri (*n* = 14).

	Total N	KCl-IN	KCl-ON	KCl-TN	Aer-IN	Aer-N_min_	Ana-N_min_	Autoclave-TN	PEIN	PEON	PETN	UV-205	UV-260	N uptake
Total N	1													
KCl-IN	0.415	1												
KCl-ON	0.412	0.240	1											
KCl-TN	0.697**	0.697**	0.700**	1										
Aer-IN	0.754**	0.714**	0.380	0.787**	1									
Aer-N_min_	0.798**	0.534*	0.363	0.737**	0.960**	1								
Ana-N_min_	0.639*	0.618*	0.486	0.867**	0.798**	0.758**	1							
Autoclave-TN	0.665**	0.640*	0.339	0.690**	0.930**	0.921**	0.753**	1						
PEIN	0.714**	0.362	0.132	0.532*	0.569*	0.569*	0.402	0.525	1					
PEON	0.108	0.055	0.286	0.160	−0.085	−0.165	−0.090	−0.305	0.103	1				
PETN	0.358	0.274	0.052	0.345	0.196	0.130	0.187	−0.029	0.490	0.824**	1			
UV-205	0.709**	0.762**	0.363	0.788**	0.943**	0.846**	0.863**	0.832**	0.516	0.374	0.314	1		
UV-260	0.557*	0.665**	0.337	0.602*	0.789**	0.670**	0.727**	0.688**	0.221	−0.037	0.068	0.873**	1	
N uptake	0.701**	0.420	0.235	0.598*	0.820**	0.868**	0.653*	0.727**	0.376	−0.024	0.218	0.749**	0.657*	1

*, *P* < 0.05

**, *P* < 0.01

KCl-IN, KCl-extractable inorganic N; KCl-ON, KCl-extractable organic N; KCl-TN, KCl-extractable total N; Aer-IN, extractable inorganic N after aerobic incubation; Aer-N_min_, potentially mineralizable N estimated by aerobic incubation; Ana-N_min_, potentially mineralizable N estimated by anaerobic incubation; PEIN, phosphate buffer-extractable inorganic N; PEON, phosphate buffer-extractable organic N; PETN, phosphate buffer-extractable total N; UV-205, UV absorbance at 205 nm in NaHCO_3_ extract; UV-260, UV absorbance at 260 nm in NaHCO_3_ extract.

The soil enzyme activities differed among the fertilization management treatments, but the variation was dependent on the enzymes (Table 3). The PR activity was most affected by fertilization, whereas the LA activity was relatively constant: the coefficients of variation, in decreasing order, were 91.9% for PR, 77.6% for UR, 41.2% for BG, 38.2% for NAG, and 8.2% for LA. Spearman’s rank correlation analysis showed that all the enzyme activities except for UR showed significant positive correlations with each other (*P* < 0.05).

**Table 3:** Soil enzyme activities in the soil used.

	BG	NAG	PR	UR	LA	NAG/BG	PR/BG	UR/BG	LA/BG
	(μmol *p*-NP g^-1^ h^-1^)		(μg tyr g^-1^ 2h^-1^)	(μg NH_4_^+^-N g^-1^ 2h^-1^)	(μmol NH_4_^+^-N g^-1^ h^-1^)				
Arable Andosols in Nagano Prefecture Vegetable and Ornamental Crops Experiment Station at Shiojiri									
No fertilizer	0.05	0.07	112	2.47	1.26	1.38	2110	46.7	23.8
PK	0.09	0.18	187	6.44	1.25	1.96	2023	69.6	13.5
NK	0.08	0.07	10.9	30.1	1.20	0.84	128	355	14.2
NP	0.14	0.11	88.5	11.0	1.34	0.79	637	79.2	9.66
NPK	0.19	0.15	272	18.1	1.34	0.77	1402	93.4	6.91
Low NPK	0.18	0.15	384	10.1	1.43	0.84	2131	56.1	7.91
High NPK	0.26	0.14	144	37.1	1.29	0.53	556	144	5.00
Very high NPK	0.18	0.11	445	12.8	1.31	0.60	2415	69.5	7.12
20 t compost ha^-1^	0.19	0.27	1120	3.61	1.51	1.41	5757	18.6	7.75
PK + 20 t compost ha^-1^	0.24	0.15	1041	9.56	1.59	0.62	4410	40.5	6.75
NK + 20 t compost ha^-1^	0.22	0.16	383	36.2	1.50	0.73	1771	167	6.94
NP + 20 t compost ha^-1^	0.29	0.22	1392	23.1	1.43	0.75	4803	79.7	4.94
NPK + 20 t compost ha^-1^	0.27	0.25	1120	8.69	1.45	0.90	4109	31.9	5.32
NPK + 40 t compost ha^-1^	0.32	0.21	1640	4.49	1.42	0.66	5132	14.1	4.44
Arable soils from other sites									
Andosols (*n* = 21)	0.41 ± 0.52	NA	NA	49.2 ± 44.2	2.55 ± 1.21	NA	NA	152 ± 126	8.56 ± 3.83
Acrisols (*n* = 3)	0.14 ± 0.01	NA	NA	13.0 ± 0.2	1.50 ± 0.05	NA	NA	91.6 ± 10.2	10.6 ± 1.1
Fluvisols (*n* = 3)	0.15 ± 0.02	NA	NA	14.6 ± 0.8	1.63 ± 0.06	NA	NA	95.6 ± 14.6	10.6 ± 1.0
Forest soils									
Cambisols (*n* = 22)	0.60 ± 0.43	NA	NA	20.8 ± 15.0	0.69 ± 0.38	NA	NA	45.4 ± 36.3	1.51 ± 0.95
Andosols (*n* = 7)	0.33 ± 0.10	NA	NA	18.3 ± 15.2	0.76 ± 0.36	NA	NA	56.8 ± 43.4	2.59 ± 1.41

BG, β-D-glucosidase; NAG, *N*-acetyl-β-glucosaminidase; PR, protease; UR, urease; LA, L-asparaginase; NA, not available.

**Fig 1:**
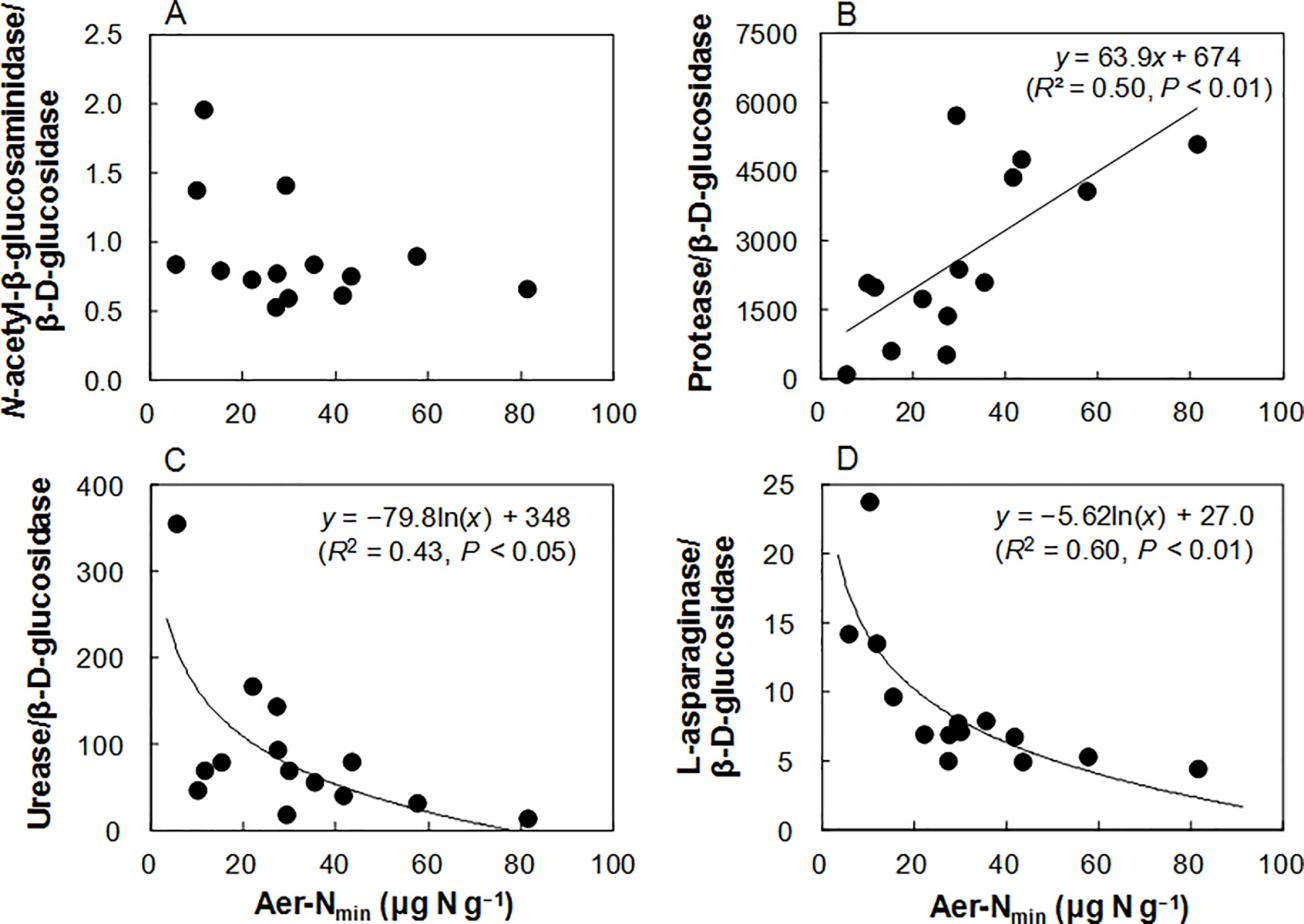
**Relationships between potentially mineralizable N estimated by aerobic incubation (Aer-N_min_) and ratios of (A) *N*-acetyl-β-glucosaminidase/β-D-glucosidase, (B) protease/β-D-glucosidase, (C) urease/β-D-glucosidase, and (D) L-asparaginase/β-D-glucosidase activities in the arable Andosols (*n* = 14) at Shiojiri**.

**Fig 2:**
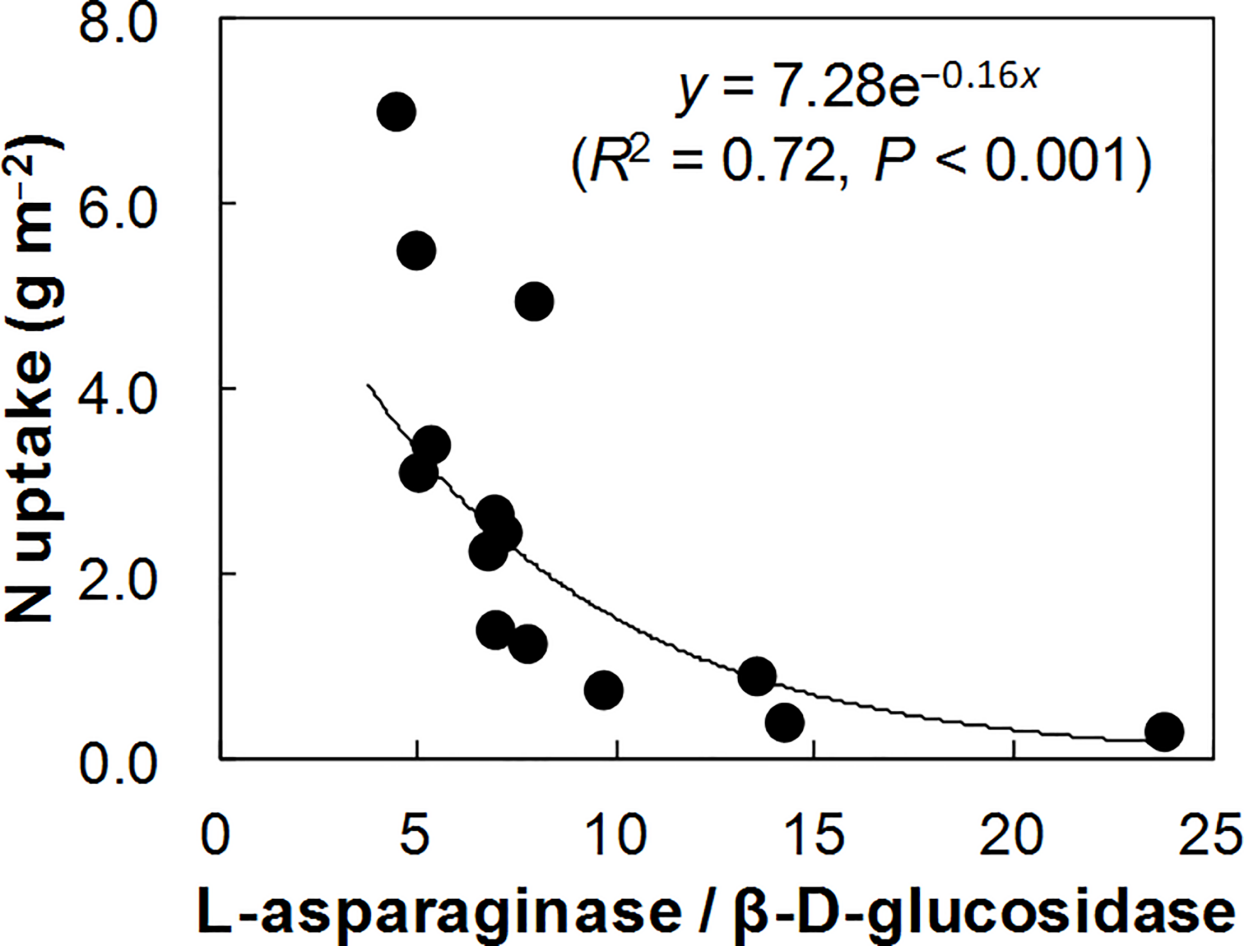
Relationship between the ratio of L-asparaginase/β-D-glucosidase activities and N uptake of celery in the arable Andosols (*n* = 14) at Shiojiri.

To test the validity of the resource allocation model for N acquiring enzymes synthesis in the Shiojiri soils, we calculated the ratios of the N acquiring enzymes to BG activities (Table 3). The LA/BG ratio increased significantly with decreases in Aer-N_min_ (*R*^2^ = 0.60, *P* < 0.01; Fig 1D), UV-260 (*R*^2^ = 0.54, *P* < 0.01), UV-205 (*R*^2^ = 0.47, *P* < 0.01), Aer-IN (*R* ^2^ = 0.46, *P* < 0.01), autoclave TN (*R*^2^ = 0.40, *P* < 0.05), Ana-N_min_ (*R*^2^ = 0.31, *P* < 0.05), and total N (*R*^2^ = 0.29, *P* < 0.05), with the strongest relationship being observed with Aer-N_min_. The LA/BG ratio was also significantly and negatively related with the N uptake of celery (*R*^2^ = 0.72, *P* < 0.001; Fig 2). The UR/BG ratio increased significantly with decreases in Aer-N_min_ (*R*^2^ = 0.43, *P* < 0.05; Fig 1C), PEIN (*R*^2^ = 0.31, *P* < 0.05), and autoclave TN (*R*^2^ = 0.29, *P* < 0.05), with the strongest relationship being observed with Aer-N_min_. However, the NAG/BG and the PR/BG ratios did not show significant negative relationships with available N and total N concentrations (*P* > 0.05). The NAG/BG ratio had a significant positive correlation with soil pH (Spearman’s *r* = 0.667, *P* < 0.01), and stepwise multiple regression analysis demonstrated simultaneous, significant effects of soil pH and Aer-N_min_ on the NAG/BG ratio as follows: NAG/BG = 5.41 × pH– 5.15 × ln(Aer-N_min_)– 9.07 (adjusted *R*^2^ = 0.67, *P* < 0.001). In contrast, the PR/BG ratio showed significant positive correlations with Aer-N_min_ (*R*^2^ = 0.50, *P* < 0.01; Fig 1B), total N (*R*^2^ = 0.46, *P* < 0.01), UV-205 (*R*^2^ = 0.45, *P* < 0.01), autoclave TN (*R*^2^ = 0.44, *P* < 0.01), Aer-IN (*R*^2^ = 0.39, *P* < 0.05), Ana-N_min_ (*R*^2^ = 0.35, *P* < 0.05), total C (*R*^2^ = 0.31, *P* < 0.05), and PEIN (*R*^2^ = 0.31, *P* < 0.05). On the basis of these results, we selected LA and UR as representative N-acquiring enzymes, and further examined the relationships between available N concentration and LA/BG and UR/BG ratios in various soils.

### Soil chemical properties and enzyme activities in various soils

We investigated whether the significant negative relationships between available N concentration and the ratios of LA/BG and UR/BG observed in Shiojiri soils held true in various arable and forest soils. At first, we evaluated the relationships for combined data for Shiojiri soils (*n* = 14) and other arable soils (*n* = 27). Aer-IN, Ana-N_min_, autoclave-TN, and UV-205 had significant positive correlations with each other in the arable soils (*P* < 0.01; S2 Table). The LA/BG ratio increased significantly with decreases in Aer-N_min_ (*R*^*2*^ = 0.37, *P* < 0.001; *n* = 41), Aer-IN (*R*^*2*^ = 0.17, *P* < 0.01; *n* = 41), autoclave-TN (*R*^*2*^ = 0.28, *P* < 0.01; *n* = 30), total N (*R*^*2*^ = 16, *P* < 0.05; *n* = 41), and Ana-N_min_ (*R*^*2*^ = 0.19, *P* < 0.05; *n* = 30), with the strongest relationship being observed with Aer-N_min_. The UR/BG ratio had no significant relationships with available N concentration and soil pH (*P* > 0.05). However, a stepwise multiple regression analysis demonstrated simultaneous, significant effects of soil pH and Aer-N_min_ on the UR/BG ratio in the arable soils as follows: UR/BG = –80.1 × pH– 39.5 × ln(Aer-N_min_)– 750.1 (adjusted *R*^2^ = 0.17, *P* < 0.05).

Next, we examined the available N concentrations and LA/BG and UR/BG ratios in forest soils (*n* = 29). When compared with the arable soils, the soil pH and LA activity was significantly lower in the forest soils (*P* < 0.01), whereas the total C, total N, Aer-IN, Aer-N_min_, Ana-N_min_, PEON, UV-205, and BG activity were significantly higher in the forest soils (*P* < 0.01; Tables 1 and 3). In the forest soils, chemical properties including soil pH, total C and available N, and UR and LA activities were not significantly different between Andosols and Cambisols (Welch–Aspin test, *P* > 0.05), whereas BG activity was significantly higher in Cambisols than in Andosols (*P* < 0.05). Significant positive correlations (*P* < 0.01) were found between available N concentration estimated by the aerobic incubation method (Aer-IN and Aer-N_min_), and the total N and Ana-N_min_, and between autoclave-TN and UV-205 in the forest soils (S3 Table). The ratios of LA/BG and UR/BG had no significant relationships with available N concentration and pH in the forest soils (*P* > 0.05).

**Fig 3:**
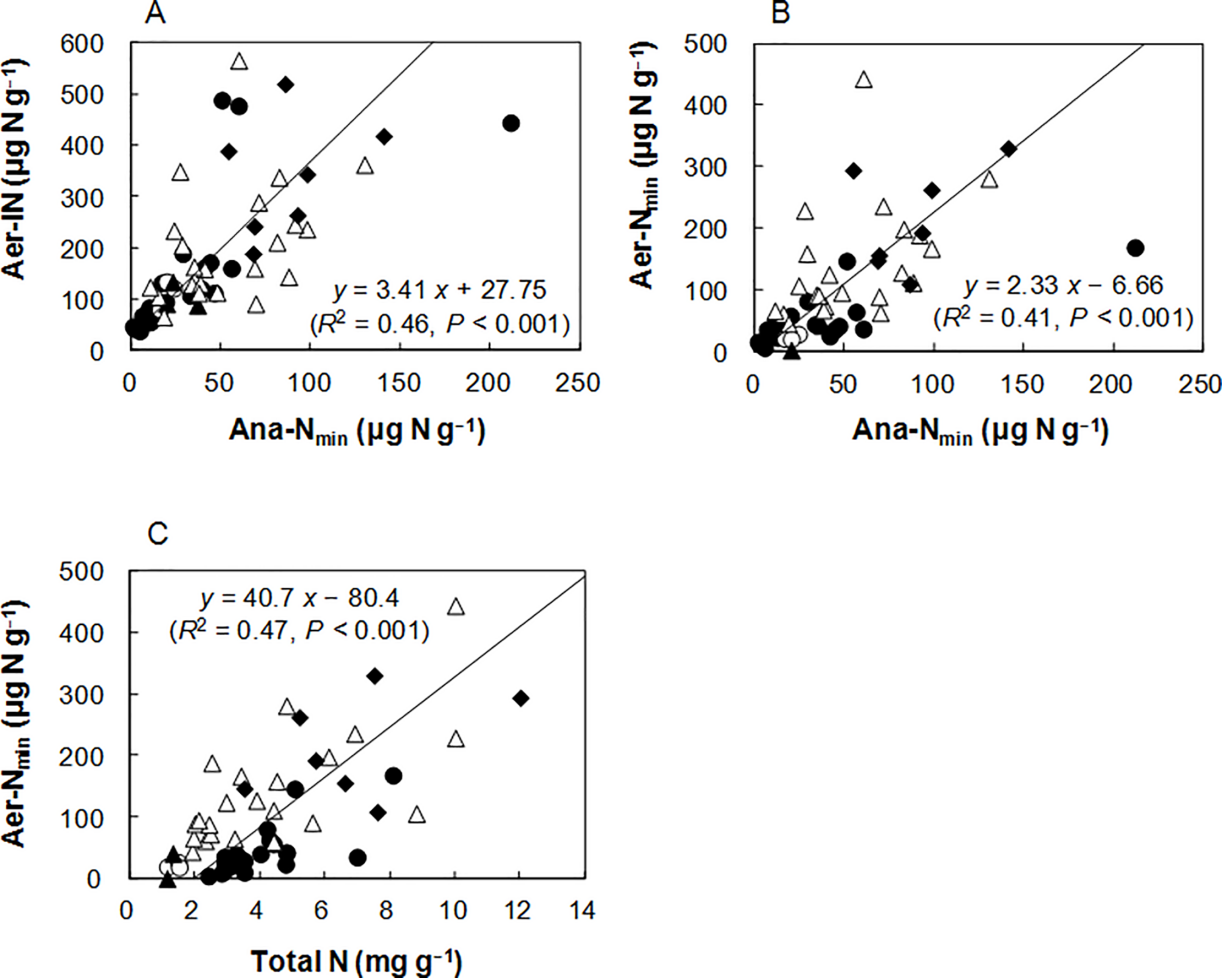
**Standardized major axis regression relationships (A) between potentially mineralizable N estimated by anaerobic incubation (Ana-N**_**min**_**) and extractable inorganic N after aerobic incubation (Aer-IN), (B) between Ana-N**_**min**_ **and potentially mineralizable N estimated by aerobic incubation (Aer-N**_**min**_**), and (C) between total N and Aer-N**_**min**_ **in arable and forest soils (*n* = 59).** For the arable soils; ●: Andosols; ○: Acrisols; ▲: Fluvisols. For the forest soils; Δ: Cambisols; ◆: Andosols.

When data were combined for the arable and forest soils (*n* = 70), in general, consistent relationships across land use were obtained between enzyme activities and also between N availability indices. All the N availability indices showed significant correlations with each other (S4 Table). In particular, available N concentration estimated by the aerobic incubation method (Aer-IN and Aer-N_min_) had significant strong relationships with Ana-N_min_ and total N (Fig 3). The BG activity had a significant negative correlation with soil pH (Spearman’s *r* = −0.401, *P* < 0.001) and positive correlations with total C (*r* = 0.398, *P* < 0.001) and total N (*r* = 0.400, *P* < 0.001), whereas LA activity showed a significant positive correlation with soil pH (*r* = 0.460, *P* < 0.001) but not with total C and total N (*P* > 0.05). The UR activity did not significantly correlate with the total C, total N, and soil pH (*P* > 0.05).

**Fig 4:**
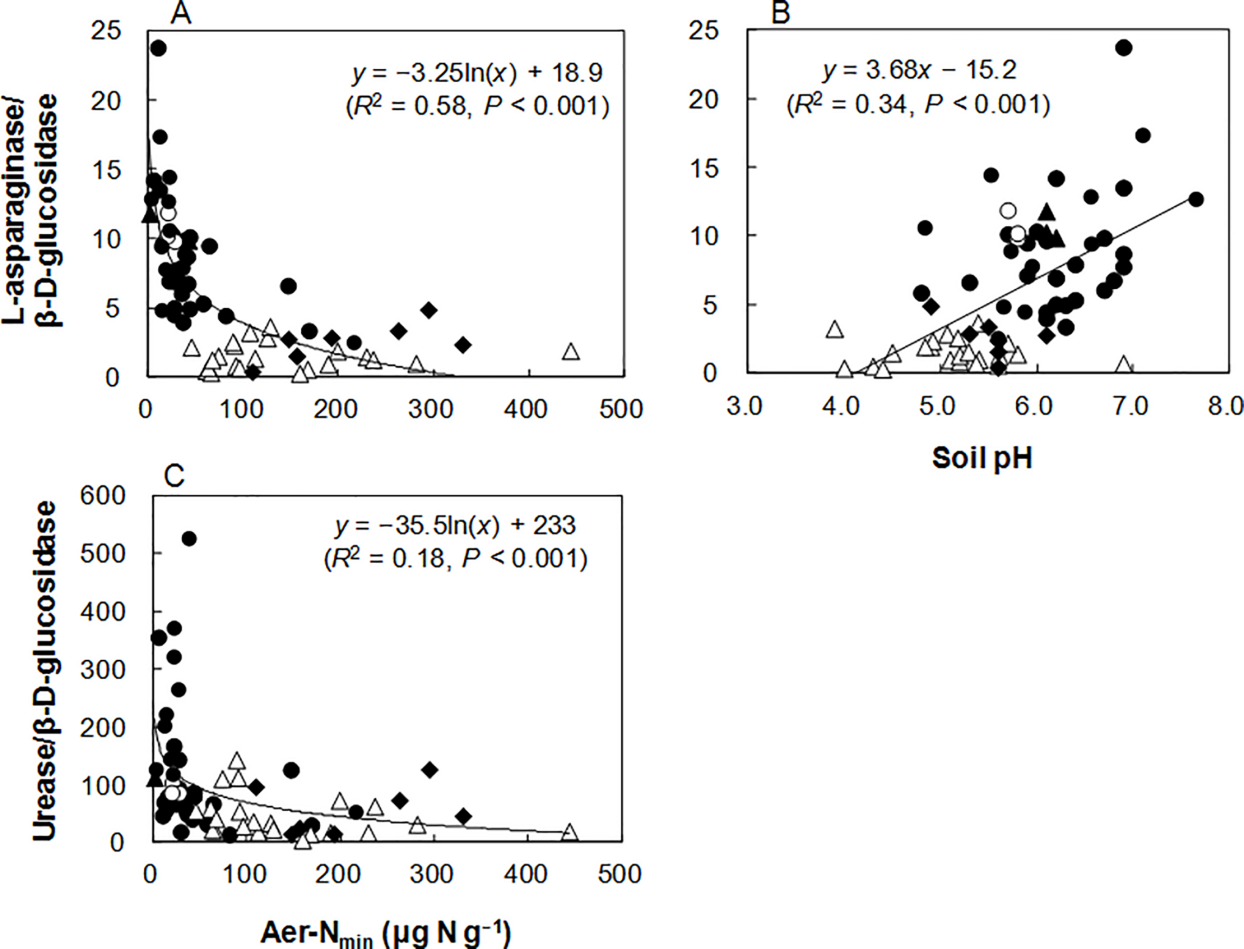
**Relationships of the ratio of L-asparaginase/β-D-glucosidase activities with (A) potentially mineralizable N estimated by aerobic incubation (Aer-N**_**min**_**) and (B) soil pH, and (C) between the ratio of urease/β-D-glucosidase activities and Aer-N**_**min**_ **in arable and forest soils (*n* = 70).** For the arable soils; ●: Andosols; ○: Acrisols; ▲: Fluvisols. For the forest soils; Δ: Cambisols; ◆: Andosols.

In the arable and forest soils, the LA/BG ratio increased significantly with decreases in Aer-N_min_ (*R*^2^ = 0.58, *P* < 0.001; Fig 4A), UV-205 (*R*^2^ = 0.40, *P* < 0.001), Ana-N_min_ (*R*^2^ = 0.35, *P* < 0.001), PEON (*R*^2^ = 0.34, *P* < 0.001), Aer-IN (*R*^2^ = 0.33, *P* < 0.001), total N (*R*^2^ = 0.16, *P* < 0.01), autoclave TN (*R*^2^ = 0.12, *P* < 0.01), UV-260 (*R*^2^ = 0.11, *P* < 0.05), and PETN (*R*^2^ = 0.11, *P* < 0.05), with the strongest relationship being observed with Aer-N_min_. The LA/BG ratio also increased significantly with soil pH (Fig 4B). The UR/BG ratio increased significantly with decreases in Aer-N_min_ (*R*^2^ = 0.18, *P* < 0.001; Fig 4C), UV-205 (*R*^2^ = 0.14, *P* < 0.01), UV-260 (*R*^2^ = 0.11, *P* < 0.01), Ana-N_min_ (*R*^2^ = 0.10, *P* < 0.05), and Aer-IN (*R*^2^ = 0.08, *P* < 0.05). A stepwise multiple regression analysis demonstrated simultaneous, significant effects of Aer-N_min_ and soil pH on the LA/BG and UR/BG ratios as follows: LA/BG = 1.49 × pH– 2.71 × ln(Aer-N_min_)– 8.11 (adjusted *R*^2^ = 0.60, *P* < 0.001); UR/BG = –25.8 × pH– 45.2 × ln(Aer-N_min_)– 420.2 (adjusted *R*^2^ = 0.19, *P* < 0.001).

There was no significant effect of the storage conditions (i.e., air-dried and rewetted vs. frozen at −20°C) on the relationships between the ratios of LA/BG and UR/BG and Aer-N_min_. Furthermore, our preliminary study showed that the LA/BG ratio was very similar when the soil sample was stored under both conditions, although the ratio was slightly higher in samples stored at −20°C than in rewetted samples that were stored air-dried, with the largest difference being 16%. Thus, the effects of soil storage conditions on the LA/BG and UR/BG ratios seemed to be small in this study.

## Discussion

According to the resource allocation model for extracellular enzyme synthesis [4], microorganisms will preferentially allocate their resources to synthesize enzymes that are involved in the acquisition of the most limiting element. However, the situation is complicated for N-acquiring enzymes, because mineralization of organic N compounds is often coupled to energy acquisition [3, 7–9], and because unlike P-acquiring phosphatase enzymes, which have wide substrate affinities, each N compound is decomposed by distinct enzyme systems, as discussed by Sinsabaugh et al. [43]. In the present study, we compared the response of synthesis of four N-acquiring enzymes to soil N availability and also examined which soil N form and/or N availability index microorganisms respond to when investing their resources in N-acquiring enzyme synthesis in arable and forest soils.

In the arable Shiojiri soils, both LA/BG and UR/BG ratios showed significant negative relationships with available N concentration, especially Aer-N_min_ (Fig 1). These results suggest that microorganisms synthesize LA and UR in response to soil N availability, in particular Aer-N_min_, in the Shiojiri soils. In contrast, both NAG/BG and PR/BG ratios did not show significant negative relationships with available N concentrations (*P* > 0.05), although simultaneous significant effects of soil pH and Aer-N_min_ on the NAG/BG ratio were observed using multiple regression analysis. It is noteworthy that the LA/BG ratio exhibited a significant negative relationship with the N uptake of celery (Fig 2) and that both N uptake of celery (Table 2) and LA/BG and UR/BG ratios were most strongly influenced by Aer-N_min_ among various indices of N availability. These results imply that microbes and celery might access the same N pools in the soils, as noted in Sullivan et al. [9].

Based on the results of the arable Shiojiri soils, we measured LA and UR as N-acquiring enzymes in various arable and forest soils and investigated the validity of the resource allocation model for LA and UR synthesis. The LA/BG and UR/BG ratios increased with decreases in some indices of N availability, with the strongest relationship being observed with Aer-N_min_ (Fig 4) followed by indices such as UV-205 and Ana-N_min_. A stepwise multiple regression analysis revealed significant effects of Aer-N_min_ and soil pH on the LA/BG and UR/BG ratios. Therefore, microbial LA and UR synthesis might be regulated primarily by N availability in the soils, but the influence of soil pH was also significant.

The relationships between the LA/BG and UR/BG ratios and the N availability (Fig 4) corresponded with the EnzOpt model [44], which optimizes the stoichiometry of resource return to match the microbial stoichiometry, suggesting that synthesis of LA and UR was optimized to acquire N, but not C, in the soils. Unlike the LA/BG and UR/BG ratios, the PR/BG and NAG/BG ratios did not show significant negative relationships with available N concentration (Fig 1). Hence, synthesis of PR and NAG might not be controlled primarily by N availability in the soils. This difference in response to soil N availability between N-acquiring enzymes could be explained by the underlying mechanism for synthesis of these enzymes in soil microorganisms. It has been reported that expression of the gene encoding LA is activated by N limitation and repressed by adding NH_4_^+^ and amino acids in the yeast *Saccharomyces cerevisiae* [45] and in the bacterium *Bacillus subtilis* [46], although intracellular LA is constitutively expressed in *S*. *cerevisiae* [45]. In *B*. *licheniformis*, LA is synthesized to acquire N but not to acquire C [47]. Atkinson and Fisher [48] reported that syntheses of both LA and UR were increased in the presence of N compounds that were poor sources of NH_4_^+^ (e.g., aspartate, proline, glutamate) but not increased either under C limitation nor in the presence of NH_4_^+^ or N compounds that were good sources of NH_4_^+^ (e.g., arginine, asparagine, glutamine) in *B*. *subtilis*. McCarty et al. [49] reported the suppression of microbial UR production by adding inorganic N or amino acids to soils. According to Mobley and Hausinger [50], microbial regulation of UR production is classified into three types: (1) the synthesis is activated under N-limiting conditions, whereas the synthesis is repressed in the presence of NH_4_^+^ or nitrogen-rich compounds including urea; (2) the synthesis is induced by the presence of the substrate (i.e., urea); and (3) the enzyme is synthesized constitutively. Contrary to the case of LA and UR, microbial synthesis of extracellular PR is induced not only by N limitation but also by C or S limitation in bacteria [51, 52] and fungi [53, 54]. Also, production of NAG is under C catabolite repression in bacteria [55] and a fungus *Penicillium chrysogenum* [56]. Considering these together, we assumed that soil microbes that produced more LA and UR when N availability was lower were predominant in LA- and UR-producing microbial communities, whereas the effects of other factors (e.g., microbial energy demand and pH) were larger than that of N availability for synthesis of NAG and PR in the soils, partly because they are also C-acquiring enzymes.

It should be noted that both LA and UR are probably present intracellularly as well as extracellularly in soils, although the resource allocation model was developed for extracellular enzyme synthesis [3, 4]. Various bacteria, filamentous fungi, and yeasts synthesize extracellular LA [57], but some microorganisms produce both intracellular and extracellular LA [45]. Most of the microbial URs appear to be cytoplasmic protein [58]; it was suggested that urea is taken up directly and hydrolyzed intracellularly in soil microorganisms, rather than mineralized extracellularly [59]. However, a significant portion of the UR activity in soils might be because of extracellular enzymes [60–62]. At present, clear experimental distinction between intracellular and extracellular enzyme activities in soils is difficult to perform [63].

Interestingly, in the present study synthesis of LA and UR was suggested to respond strongly to potentially mineralizable N (i.e., Aer-N_min_ and Ana-N_min_) but not to labile inorganic (i.e., KCl-IN) and organic (i.e., KCl-ON and PEON) N pools, whereas microorganisms synthesize phosphatase in response to the pool size of labile inorganic P in soils [5, 6]. Binkley and Vitousek [27] suggested that although extractable NH_4_^+^ and NO_3_^−^ represent the easily available inorganic forms of N in soils, the sizes of these pools are generally small relative to annual N supply rate, and they should turnover rapidly. Thus, extractable NH_4_^+^ and NO_3_^−^ only represent the available concentration at a single point of time and are poor indices of the fluxes of these ions [64]. In contrast, the Aer-N_min_ (mineralized N during 28-day aerobic incubation) and Ana-N_min_ (mineralized N during 7-day anaerobic incubation) might reflect the N supply rate in soils. We assume that microorganisms synthesize LA and UR in response to N supply rate, rather than to pool sizes of labile N (e.g., NH_4_^+^, NO_3_^−^) in the soils.

**Fig 5:**
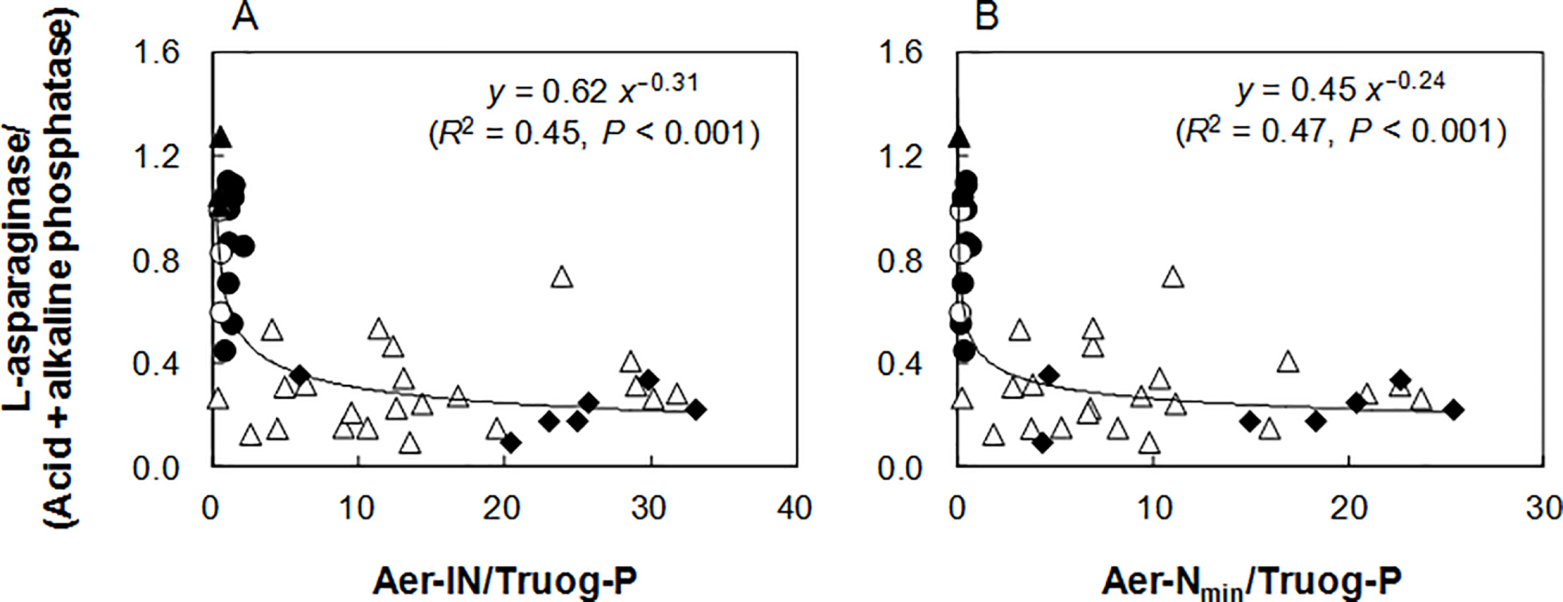
**Relationships of the ratio of L-asparaginase to acid phosphatase + alkaline phosphatase activities with (A) Aer-IN/Truog-P and (B) Ana-N**_**min**_**/Truog-P (*n* = 43).** For the arable soils; ●: Andosols; ○: Acrisols; ▲: Fluvisols. For the forest soils; Δ: Cambisols; ◆: Andosols.

To assess the effect of soil P availability on the resource allocation for N-acquiring enzyme synthesis, we cited the data of phosphatase activity (combined acid and alkaline phosphatases; ACP + ALP) and P concentrations in the arable and forest soils from Fujita et al. [6] (*n* = 43), which were also used in the present study, and analyzed whether the ratio of N-acquiring enzyme to P-acquiring enzyme activities was related to the available N/P ratio. The LA/(ACP + ALP) ratio had significant negative relationships with Aer-IN/available P (Truog-P: *R*^2^ = 0.45, *P* < 0.001, Fig 5A; H_2_O-Pi: *R*^2^ = 0.31, *P* < 0.001) and Aer-N_min_/available P (Truog-P: *R*^2^ = 0.47, *P* < 0.001, Fig 5B; H_2_O-Pi: *R*^2^ = 0.38, *P* < 0.001) in the soils. The UR/(ACP + ALP) ratio was not significantly correlated with available N/P ratio in the soils. These results suggest that microorganisms allocate their resources to LA and phosphatase synthesis in response to N and P availabilities in the soils. Although the existing forms of N and P (i.e., availability) were not accounted for, weak but significant negative relationships were also reported between LAP/ALP ratio and total N/total P ratio in calcareous wetland soils [65], and between peptidase/phosphatase ratio and total N/total P ratio in coastal wetlands within the Great lakes [66].

## Conclusions

The LA/BG and UR/BG ratios showed the most significant negative relationships with Aer-N_min_ and to a lesser extent with Ana-N_min_ and UV-205 but not with pool sizes of labile inorganic N in the soils, suggesting that soil microorganisms might allocate their resources to LA and UR synthesis in response to N supply rate. That is, it is the rate of N supply that matters most for microorganisms, rather than the size of the most available pools (i.e., extractable NH_4_^+^ and NO_3_^−^) at a given time, as also noted in Sullivan et al. [9]. Unlike the LA/BG and UR/BG ratios, the PR/BG and NAG/BG ratios did not show significant negative relationships with available N concentration in the soils. Hence, N-acquiring enzymes that release N compounds that cannot be taken up directly by microbial cells seem insensitive to microbial N limitation status, whereas N-acquiring enzymes that release low-molecular-weight N compounds (i.e., NH_4_^+^ and amino acids), which is taken up directly by microorganisms, seem to be induced by N-limitation status of the microbial community. This variation in response to soil N availability between N-acquiring enzymes might be because of the difference in the underlying mechanism for synthesis of these enzymes in soil microorganisms: probably, microbial LA and UR syntheses were primarily regulated by N availability, whereas the effects of other factors other than N availability mainly determined NAG and PR syntheses in the soils. To our knowledge, this is the first report demonstrating the strong link between the rate of N supply and the microbial resource allocation for synthesis of N-acquiring enzymes across various soils.

Strong negative relationships between the LA/BG ratio and the potentially mineralizable N in the arable and forest soils and also between the LA/BG ratio and the N uptake of celery in Shiojiri soils suggest that the resource allocation model for LA synthesis might be useful for evaluating the availability of N for both soil microorganisms and plants. If this is true, the LA/BG ratio could be a useful alternative for assessing available N level for plants as well as microorganisms in soils, because a measurement of potentially mineralizable N is time-consuming. Further studies are warranted to clarify this issue.

## Supporting information

S1 Table The fertilizer management systems in the arable Andosols at Nagano Prefecture Vegetable and Ornamental Crops Experiment Station at Shiojiri. (DOCX)

S2 Table Spearman's rank correlation coefficients (r) among various indices of N availability in arable soils (n = 41). (DOCX)

S3 Table Spearman's rank correlation coefficients (r) among various indices of N availability in forest soils (n = 29). (DOCX)

S4 Table Spearman's rank correlation coefficients (r) among various indices of N availability in arable and forest soils (n = 70). (DOCX)

S1 Fig **Representative data for the effect of rewetting of air-dried soil on its (a) β-D-glucosidase (BG) and (b) L-asparaginase (LA) activities, and (c) their ratio (LA/BG)**.
(PDF)
