# Supplementary material for: Nitrogen supply rate regulates microbial resource allocation for synthesis of nitrogen-acquiring enzymes
Source: PLoS One. 2018 Aug 14;13(8):e0202086. doi: 10.1371/journal.pone.0202086 (PMC6091965; doi:10.1371/journal.pone.0202086)
Supplement: S1 Table — (DOCX) [file pone.0202086.s001.docx]

| **S1 Table. The fertilizer management systems in the arable Andosols at Nagano Prefecture Vegetable and Ornamental Crops Experiment Station at Shiojiri** | | | | | | | |
| --- | --- | --- | --- | --- | --- | --- | --- |
|  | Organic fertilizer (kg ha^−1^ yr^−1^) | | |  | Chemical fertilizer (kg ha^−1^ yr^−1^) | | |
|  | N | P | K |  | N | P | K |
| No fertilizer | 0 | 0 | 0 |  | 0 | 0 | 0 |
| PK | 0 | 0 | 0 |  | 0 | 44 | 83 |
| NK | 0 | 0 | 0 |  | 100 | 0 | 83 |
| NP | 0 | 0 | 0 |  | 100 | 44 | 0 |
| NPK | 0 | 0 | 0 |  | 100 | 44 | 83 |
| Low NPK | 0 | 0 | 0 |  | 28 | 35 | 0 |
| High NPK | 0 | 0 | 0 |  | 172 | 55 | 192 |
| Very high NPK | 0 | 0 | 0 |  | 244 | 65 | 302 |
| 20 t compost ha^-1^ | 72 | 10 | 109 |  | 0 | 0 | 0 |
| PK + 20 t compost ha^-1^ | 72 | 10 | 109 |  | 0 | 44 | 83 |
| NK + 20 t compost ha^-1^ | 72 | 10 | 109 |  | 100 | 0 | 83 |
| NP + 20 t compost ha^-1^ | 72 | 10 | 109 |  | 100 | 44 | 0 |
| NPK + 20 t compost ha^-1^ | 72 | 10 | 109 |  | 100 | 44 | 83 |
| NPK + 40 t compost ha^-1^ | 144 | 19 | 218 |  | 100 | 44 | 83 |
