# Supplementary material for: Nitrogen supply rate regulates microbial resource allocation for synthesis of nitrogen-acquiring enzymes
Source: PLoS One. 2018 Aug 14;13(8):e0202086. doi: 10.1371/journal.pone.0202086 (PMC6091965; doi:10.1371/journal.pone.0202086)
Supplement: S2 Table — (DOCX) [file pone.0202086.s002.docx]

| **S2 Table. Spearman's rank correlation coefficients (*r*) among various indices of N availability in arable soils (*n* = 41)** | | | | | | | | | |
| --- | --- | --- | --- | --- | --- | --- | --- | --- | --- |
|  | Total N | Aer-IN | Aer-N_min_ | Ana-N_min_ | Autoclave-TN | PEON | PETN | UV-205 | UV-260 |
| Total N | 1 |  |  |  |  |  |  |  |  |
| Aer-IN | 0.390* | 1 |  |  |  |  |  |  |  |
| Aer-N_min_ | 0.513** | 0.569** | 1 |  |  |  |  |  |  |
| Ana-N_min_ | 0.391* | 0.898** | 0.609** | 1 |  |  |  |  |  |
| Autoclave-TN | 0.562** | 0.911** | 0.636** | 0.818** | 1 |  |  |  |  |
| PEON | 0.476** | 0.298 | 0.157 | 0.277 | 0.407* | 1 |  |  |  |
| PETN | 0.367* | 0.768** | 0.262 | 0.673** | 0.747** | 0.718** | 1 |  |  |
| UV-205 | 0.143 | 0.892** | 0.370 | 0.817** | 0.777** | 0.208 | 0.738** | 1 |  |
| UV-260 | −0.000 | 0.635** | 0.211 | 0.567** | 0.412* | −0.004 | 0.418* | 0.788** | 1 |
| *, *P* < 0.05; **, *P* < 0.01 | |  |  |  |  |  |  |  |  |
| (*n* = 30 for correlations with Ana-N_min_, Autocalve-TN, PEON, PETN, UV-205, and UV-260) | | | | | | | | | |
