# Supplementary material for: Nitrogen supply rate regulates microbial resource allocation for synthesis of nitrogen-acquiring enzymes
Source: PLoS One. 2018 Aug 14;13(8):e0202086. doi: 10.1371/journal.pone.0202086 (PMC6091965; doi:10.1371/journal.pone.0202086)
Supplement: S3 Table — (DOCX) [file pone.0202086.s003.docx]

| **S3 Table. Spearman's rank correlation coefficients (*r*) among various indices of N availability in forest soils (*n* = 29).** | | | | | | | | | |
| --- | --- | --- | --- | --- | --- | --- | --- | --- | --- |
|  | Total N | Aer-IN | Aer-N_min_ | Ana-N_min_ | Autoclave-TN | PEON | PETN | UV-205 | UV-260 |
| Total N | 1 |  |  |  |  |  |  |  |  |
| Aer-IN | 0.784** | 1 |  |  |  |  |  |  |  |
| Aer-N_min_ | 0.695** | 0.893** | 1 |  |  |  |  |  |  |
| Ana-N_min_ | 0.189 | 0.554** | 0.589** | 1 |  |  |  |  |  |
| Autoclave-TN | 0.345 | 0.290 | 0.201 | −0.296 | 1 |  |  |  |  |
| PEON | 0.401* | 0.526* | 0.404* | 0.373* | 0.219 | 1 |  |  |  |
| PETN | 0.463* | 0.624** | 0.457* | 0.392* | 0.320 | 0.933** | 1 |  |  |
| UV-205 | 0.252 | 0.371* | 0.285 | 0.189 | 0.536** | 0.292 | 0.401* | 1 |  |
| UV-260 | 0.412* | 0.417* | 0.478** | 0.190 | 0.467* | 0.195 | 0.185 | 0.767** | 1 |
| *, *P* < 0.05; **, *P* < 0.01 | |  |  |  |  |  |  |  |  |
