# Supplementary material for: Nitrogen supply rate regulates microbial resource allocation for synthesis of nitrogen-acquiring enzymes
Source: PLoS One. 2018 Aug 14;13(8):e0202086. doi: 10.1371/journal.pone.0202086 (PMC6091965; doi:10.1371/journal.pone.0202086)
Supplement: S4 Table — (DOCX) [file pone.0202086.s004.docx]

| **S4 Table. Spearman's rank correlation coefficients (*r*) among various indices of N availability in arable and forest soils (*n* = 70)** | | | | | | | | | |
| --- | --- | --- | --- | --- | --- | --- | --- | --- | --- |
|  | Total N | Aer-IN | Aer-N_min_ | Ana-N_min_ | Autoclave-TN | PEON | PETN | UV-205 | UV-260 |
| Total N | 1 |  |  |  |  |  |  |  |  |
| Aer-IN | 0.576** | 1 |  |  |  |  |  |  |  |
| Aer-N_min_ | 0.539** | 0.765** | 1 |  |  |  |  |  |  |
| Ana-N_min_ | 0.416** | 0.814** | 0.778** | 1 |  |  |  |  |  |
| Autoclave-TN | 0.408** | 0.603** | 0.429** | 0.323* | 1 |  |  |  |  |
| PEON | 0.550** | 0.705** | 0.720** | 0.625** | 0.413** | 1 |  |  |  |
| PETN | 0.505** | 0.811** | 0.606** | 0.633** | 0.616** | 0.829** | 1 |  |  |
| UV-205 | 0.298* | 0.761** | 0.607** | 0.629** | 0.616** | 0.600** | 0.711** | 1 |  |
| UV-260 | 0.223 | 0.549** | 0.402** | 0.403** | 0.438** | 0.280* | 0.385** | 0.775** | 1 |
| *, *P* < 0.05; **, *P* < 0.01 | |  |  |  |  |  |  |  |  |
| (*n* = 59 for correlations with Ana-N_min_, Autocalve-TN, PEON, PETN, UV-205, and UV-260) | | | | | | | | | |
