# Supplementary figures and images for: Nitrogen supply rate regulates microbial resource allocation for synthesis of nitrogen-acquiring enzymes
Source: PLoS One. 2018 Aug 14;13(8):e0202086. doi: 10.1371/journal.pone.0202086 (PMC6091965; doi:10.1371/journal.pone.0202086)

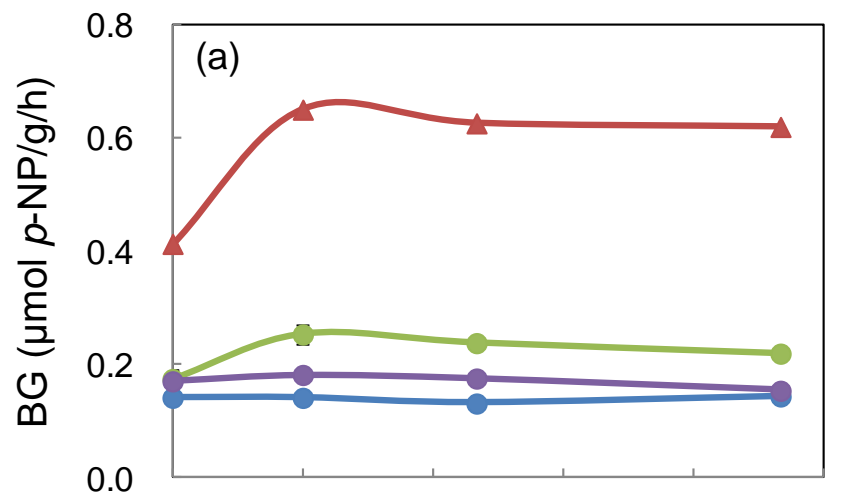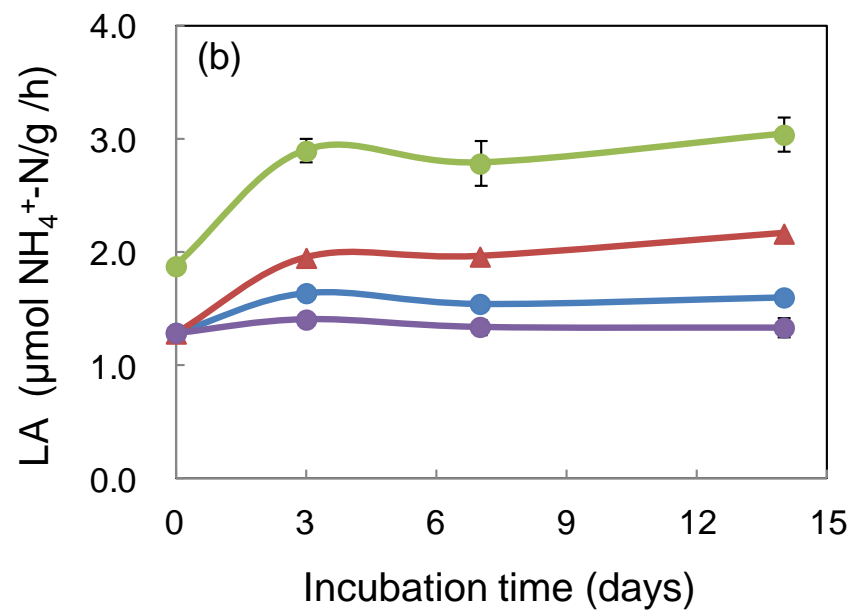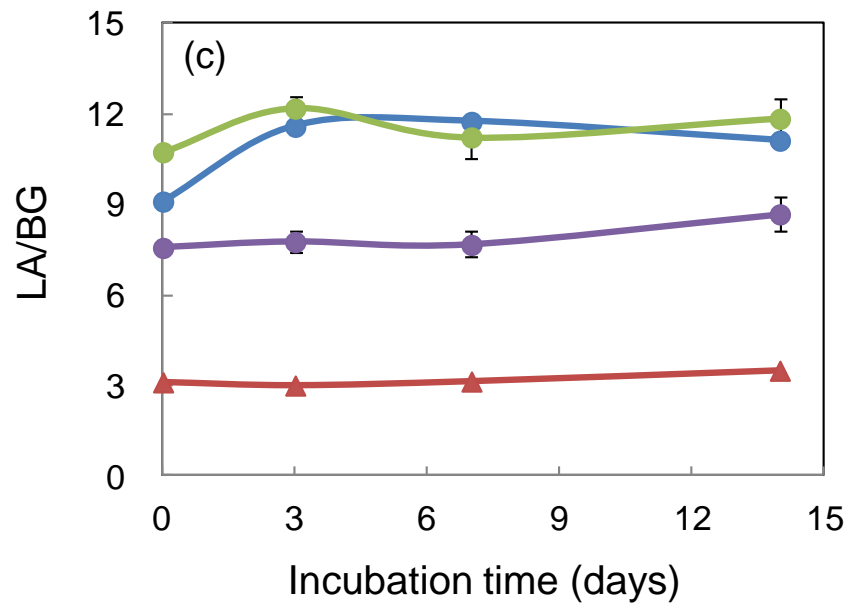

- Arable soil 1
- Arable soil 2
- Arable soil 3
- Forest soil 1

Supplement: S1 Fig — Representative data for the effect of rewetting of air-dried soil on its (a) β-D-glucosidase (BG) and (b) L-asparaginase (LA) activities, and (c) their ratio (LA/BG). (PDF) [file pone.0202086.s005.pdf]
